# Factors associated with the mental health status of pregnant women in China: A latent class analysis

**DOI:** 10.3389/fpubh.2022.1017410

**Published:** 2023-01-10

**Authors:** Yifei Pei, Qian Chen, Ying Zhang, Chenlu He, Jingjing Wang, Jie Tang, Hao Hou, Ziqing Zhu, Xunbao Zhang, Wei Wang

**Affiliations:** ^1^School of Public Health, Xuzhou Medical University, Xuzhou, Jiangsu, China; ^2^Huai'an Center for Disease Control and Prevention, Huai'an, Jiangsu, China; ^3^Wuxi No.5 People's Hospital, Wuxi, Jiangsu, China; ^4^School of Public Health, Wuhan University, Wuhan, China; ^5^Xuzhou Maternity and Child Health Care Hospital, Xuzhou, Jiangsu, China; ^6^Key Laboratory of Human Genetics and Environmental Medicine, Xuzhou Medical University, Xuzhou, Jiangsu, China

**Keywords:** pregnant women, mental health, latent class analysis, China, psychological symptoms

## Abstract

**Background:**

Prenatal mental health is a neglected public health issue that places pregnant women at a higher risk for mental disorders. The purpose of this study was to investigate the influencing factors of prenatal mental disorders and provide a scientific basis to guide and promote the mental health of pregnant women.

**Methods:**

The study sample comprised 973 women in their first pregnancy, who were in their second trimester and third trimester, who underwent obstetric outpatient checkups at the Maternal and Child Health Hospital in Huai'an, who were recruited in the survey that was conducted from July to December 2017. The Chinese mental health scale (CMHS) was used to assess the mental health of pregnant women. The present study uses the chi-square test to compare the rates of class with different demographic variables, a latent class analysis to identify psychological symptoms, and multiple logistic regression analysis to examine whether the demographics predicted class membership.

**Results:**

The chi-square test results showed that participants who reported feeling different in the perinatal period (χ^2^ = 6.35, *P* = 0.04), having marital satisfaction (χ^2^ = 15.8, *P* < 0.001), with an in-law relationship (χ^2^ = 29.43, *P* < 0.001), with a friend relationship (χ^2^ = 24.81, *P* < 0.001), with basic diseases (χ^2^ = 8.04, *P* = 0.02), and taking birth control pills (χ^2^ = 8.97, *P* = 0.01) have different probabilities of being classified. Three latent classes were identified: the high symptoms group (6.89%), the moderate symptoms group (20.56%), and the low symptoms group (72.56%). Pregnant women in the third trimester [odds ratio (OR) = 1.83, 95% confidence interval (CI): 1.04–3.25, *P* = 0.04], with a poor in-law relationship (OR = 2.82, 95% CI:1.45–5.51, *P* = 0.002), with a bad friend relationship (OR = 3.17, 95% CI: 1.31–7.71, *P* = 0.01), and who had basic diseases (OR = 1.70, 95% CI: 1.00–2.90, *P* = 0.04) tended to be classified under the high symptoms group than under the low symptoms group. Pregnant women with a bad friend relationship (OR = 2.15, 95% CI: 1.08–4.28, *P* = 0.03) and taking birth control pills (OR = 1.51, 95% CI: 1.08–2.11, *P* = 0.02) were more likely to be placed under the moderate symptoms group than under the low symptoms group.

**Conclusions:**

A pregnant woman's mental health status factors include feeling different in the perinatal period, those with marital satisfaction, those with an in-law relationship, those with a friend relationship, those with basic diseases, and those taking birth control pills. To ensure a smooth progress of pregnancy and promote the physical and mental health of pregnant women, psychological screening and psychological intervention measures should be strengthened.

## Introduction

People's material living standards and education level have gradually improved; however, the fertility rate does not seem to be optimistic in both developing countries and the least developed countries (1, 2). In this case, the health of pregnant women deserves more attention. However, about 9%−19% of pregnant women suffer from antenatal negative emotion (3), which affects their health.

In recent years, with the improvement in and popularization of medical treatment, the public's viewpoint on pregnant women has changed from purely physical health to more psychological health (4). Both living environment and social situations contribute to anxiety and depression during pregnancy (5). Gestation of life requires the mother to constantly adjust the internal environment for the embryo (6), including rising body weight and dramatically changing hormone and metabolic levels (7–9). Foremost among these, the perinatal status is a susceptible period with a high incidence of antenatal depression, which is related to maternal mortality, in particular suicide (10).

Unlike postnatal depression as a common cognitive in public, prenatal psychological problems seem to be ignored, which serve as an important cause of postnatal depressive symptoms (11). The harm inflicted by psychological problems is inestimable. It will not only make pregnant women lose control of their emotions, as under the influence of bad emotions, the suicidal tendency of pregnant women will increase (12); it will also affect their physical health as women experiencing depression or anxiety are more likely to suffer from gestational hypertension in pregnancy compared to their non-depressed or non-anxious counterparts (13). Recent evidence proved that, when a pregnant woman is exposed to depression, the development of the fetus can be affected (14), the offspring could suffer from infantile autism due to neurodevelopmental disorder (15, 16), and the children could experience worse socioemotional outcomes at the age of 6–7 years (17). For a family, it will undoubtedly cause huge financial losses apart from the spiritual burden.

There are many opinions about what can trigger prenatal psychological adversity, for example, whether hormone fluctuations during the perinatal periods lead to changes in inflammatory factors that increase the risk of depression (18). The state and personal behavior of pregnant women can also contribute to depression, such as the presence of anxiety and diseases (9, 19); smoking (20) and excessive consumption of alcohol (21); and the education level and work status (housewife/unemployment (2)). In addition, social support plays an important role (22), especially when the pregnant woman receives inadequate support from the husband (domestic violence (23)) or friends and lives in a disturbed family environment (24); the choice of induction of labor (25); etc.

While the factors responsible for prenatal mental disorders are complex, it is also recognized that pregnant women at risk of prenatal mental disorder are heterogeneous. Through observed and measurable behavior, the latent mental health of pregnant women can be indirectly measured in studies on their mental health. In previous studies, the standard for classifying the mental health of pregnant women was based on the sum of the scores of self-assessment scales (26, 27). The categorization standard was too straightforward to discriminate between group traits. The application of latent class analysis (28) (LCA) can solve this problem and classify the potential characteristics of a population based on the score probability of each item. Latent class analysis (28) (LCA) is an analytical method for locating homogeneous groups (classes) of people. Despite the fact that class membership is unknown, it can be inferred from response patterns to a set of categorical variables.

Due to the traditional thought of a feudal society, prenatal mental disorders have been less focused by the Chinese family. The purpose of this study was to use the LCA method to investigate the influencing factors of prenatal mental disorders to explore the variation from perinatal pregnant women in different groups and to provide a scientific basis for reducing prenatal mental disorders.

## Materials and methods

### Participants and procedures

In the present study, the participants were pregnant women in their first pregnancy, who were in the second trimester and third trimester, who underwent obstetric outpatient checkups at the Maternal and Child Health Hospital in Huai'an.

The inclusion criteria were as follows: Pregnant women who underwent antenatal checkups at our hospital and were interested in taking part in this study. The exclusion criteria were as follows: (1) women who were suffering from psychiatric and other psychotic disorders; (2) women who were experiencing serious physical diseases; (3) women who could not cooperate with the investigation owing to education level or other reasons; and (4) women who were refusing to participate.

Finally, a total of 1,000 participants were interviewed by the questionnaire from July to December 2017. After excluding individuals with missing data for key variables, 973 participants were used for analysis (response rate: 97.3%). Ethical approval for this study was obtained from the Medical Ethics Committee of Xuzhou Medical University and the Maternal and Child Health Hospital in Huai'an. The procedures used in this study adhere to the tenets of the Declaration of Helsinki. All participants gave their written informed consent before their inclusion in the present study.

### Measures

The questionnaire included sociodemographic information such as age (≤ 25/26–34/≥35), education (high school or less/universities and colleges/undergraduate/postgraduate), being an only child (Yes/No), residence (urban/rural), monthly household income (< 3,000/3,000–5,000/5,001–7,000/>7,000 RMB), perinatal period (second trimester/third trimester), number of marriages (one marriage/two marriages and above), marital satisfaction (satisfied/dissatisfied), conjugal relation (good/bad), husband and wife status (equality/inequality), in-law relationship (good/bad), friend relationship (good/bad), basic diseases (Yes/No), and taking birth control pills (Yes/No).

The Chinese mental health scale (29) (CMHS) is one of the most extensively used tools for mental health screening. The CMHS consists of 10 subscales: (1) interpersonal tension; (2) poor psychological endurance; (3) poor adaptability; (4) psychological imbalance; (5) emotional disorder; (6) anxiety; (7) depression; (8) hostility; (9) stubbornly biased; and (10) somatization. Each subscale consists of eight items. On a 5-point Likert scale, the participants rate items from 1 (not at all) to 5 (nearly every day). The problem severity of each subscale is classified as none (average score of scale < 2), mild (2–2.99), moderate (3–3.99), and severe (≥4). The sum of the scores of each of the 80 items divided by 80 is the total average score of mental health. The subscale indicates whether there are problems in 10 aspects of the mental health of the subjects, and the total average score of the CMHS evaluates the overall mental health of the subjects. The total average score ranges from 1 to 5. The severity of mental health problems is categorized as no mental health problems (average score of scale < 2), mild mental health problems (2–2.99), moderate mental health problems (3–3.99), and severe mental health problems (≥4). Cronbach's alpha for CMHS was 0.97 and Cronbach's alpha for each subscale was above 0.85 in this study.

### Statistical analysis

The data was collected through the Epidata 3.1 database and analyzed in SPSS 23.0 and Mplus (Mplus Version 7.4). Data analysis consisted of three parts. First, the chi-square test was performed to compare the rate of class with different demographic variables. Afterward, a latent class analysis was applied to identify psychological symptoms.

The 10 components of the CMHS were dichotomized as present or absent (values of 1 or 0 as categorical indicator variables). Latent class analysis (LCA) is a methodological approach that helps to explain population heterogeneity within observed data through the identification of underlying subgroups of individuals, thus allowing the examination of different HRBs while dealing with a diverse nature of the population (30). Six model fit indices were used to assist in determining the best model for LCA: Akaike Information Criterion (AIC), Bayesian Information Criterion (BIC), adjusted Bayesian Information Criterion (aBIC), Lo-Mendell-Rubin (LMR), Bootstrapped Likelihood Ratio Test (BLRT), and Entropy (31). LMR and BLRT are used to make a comparison between the estimated model and a model with *k*-1 class, or classes, with k equaling the number of classes (32). A low and significant *P*-value for the LMR and BLRT indicates that the estimated model outperforms the model with one fewer class (32). The AIC, BIC, and aBIC are frequently used to compare different counterpart models, with the lowest value for each indicator indicating the best-fitting model (33).

Finally, multiple logistic regression analysis was used to examine whether the demographics predicted class membership.

## Results

### Latent class analysis of 10 psychological symptoms

**Figure 1:**
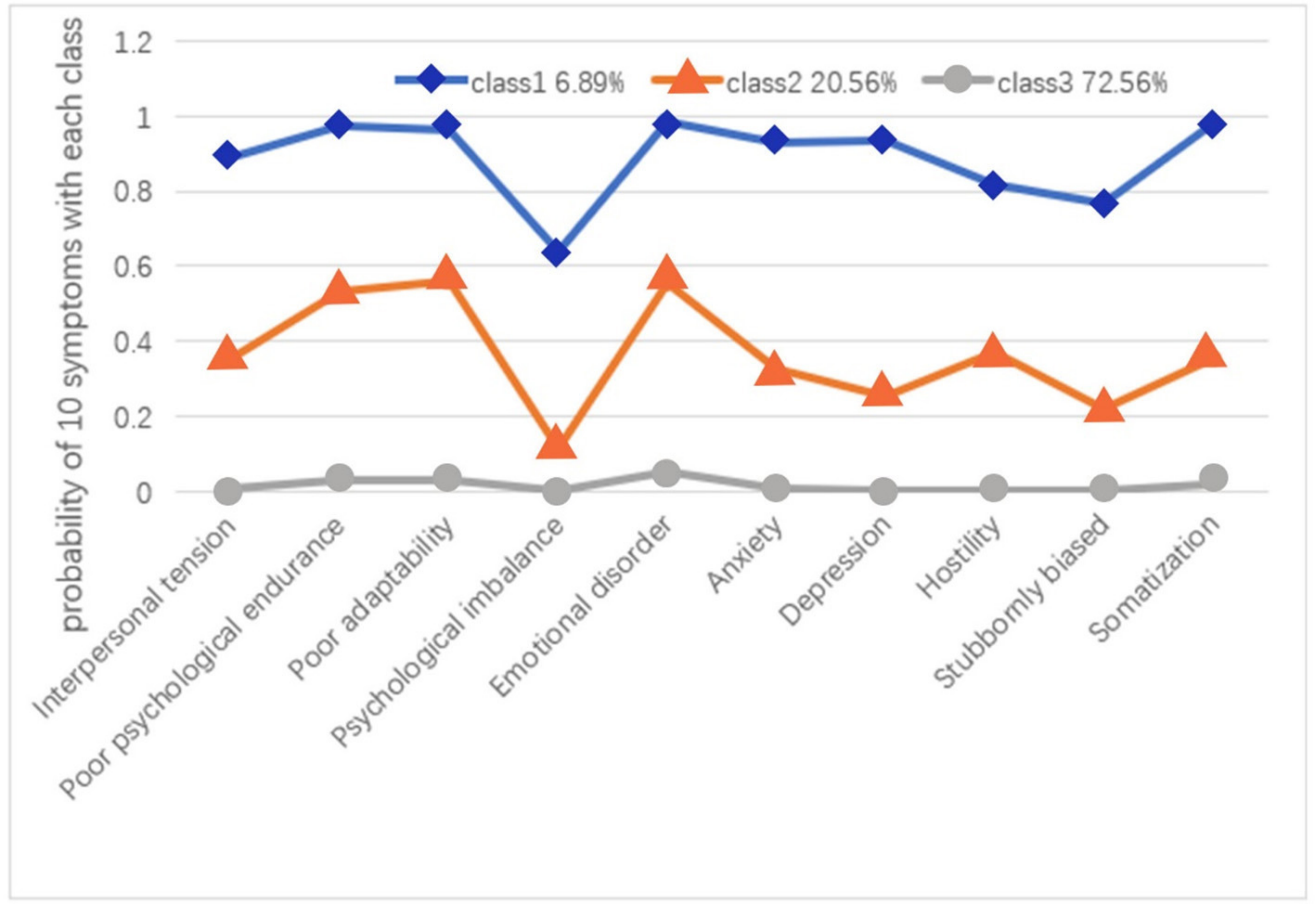
The three-class model and probability of 10 psychological symptoms within each class (*n* = 973). Class 1: the high symptoms group, Class 2: the moderate symptoms group, Class 3: the low symptoms group.

Patterns of psychological symptoms were identified by the LCA in Mplus (Mplus Version 7.4) to the five classes. The five-class and four-class models did not replicate the best LMR-LRT and BLRT values and were therefore not considered further. The three latent classes were regarded as the best model based on a series of indices (Table 1), which showed the lowest AIC, BIC, and aBIC values. Furthermore, the *P*-values of LMR-LRT and BLRT were statistically significant in class 3. Although, *P*-values of LMR-LRT and BLRT test were significant for both 2-class and 3-class models, the lower AIC, BIC, and aBIC values for the 3-class model indicated better fitness of this model in the present study. In this study, we chose the 3-class solution because it had the lowest BIC, AIC, aBIC, and LMR-LRT < 0.0001. Figure 1 shows the three identified latent classes. Latent class 1 was named as a “high symptoms group” that includes 6.89% of the subjects (*n* = 973). In this class, pregnant woman reported high probabilities of all 10 psychological symptoms, especially in regard to poor psychological endurance, emotional disorder, and somatization. Class 2 (moderate symptom group, 20.56% of subjects) showed high probabilities of poor psychological endurance, poor adaptability, and emotional disorder. Class 3 (72.56% of subjects) was characterized by the lowest probabilities for all 10 psychological symptoms and was therefore named the “low symptoms group.”

**Table 1:** Model fit indices derived from latent class analysis on models with 1–5 classes.

**Model**	**k**	**AIC**	**BIC**	**aBIC**	**Entropy**	**LMR**	**BLRT**	**Class probability**
1	10	7,978.61	8,027.41	7,995.65				
2	21	5,513.19	5,615.68	5,548.99	0.95	< 0.001	< 0.001	208/765 (0.21/0.79)
**3**	**32**	**5,205.72**	**5,361.90**	**5,260.26**	**0.92**	**< 0.001**	**< 0.001**	**67/200/706 (0.07/0.20/0.73)**
4	43	5,186.05	5,395.91	5,259.34	0.89	0.23	< 0.001	164/700/44/65 (0.17/0.72/0.05/0.07)
5	54	5,173.52	5,437.06	5,265.56	0.90	0.51	< 0.001	30/152/48/703/40 (0.03/0.16/0.05/0.72/0.04)

AIC, Akaike information criterion; BIC, Bayesian information criterion; aBIC. sample size adjusted BIC; LMR, Vuong-Lo-Mendell-Rubin likelihood ratio test; BLRT, bootstrapped likelihood ratio test. The three latent classes were regarded as the best model, highlighted in bold.

### The rate of three classes with different demographic variables

The participants' demographic characteristics of the three classes are listed in Table 2, the mean age of the participants was 28.24 years. Most of them had completed at least a high school education, and 73.48% of them were not the only child to their parents. Approximately 70.00% lived in urban areas, their monthly household income was more than 7,000 RMB, accounting for 37.83%. In total, 94.35% of the sample reported one marriage and 59.20% were in the third trimester at the time of investigation. There were 790 people who were satisfied with their marriage, and most of the participants reported good relation and equal status in couples. In addition, over 80.00% of them had good in-law relationship and friend relationship, 67.63% had no basic diseases, and 64.75% of the participants did not take birth control pills.

**Table 2:** Classification and comparison of three latent phenotypes of pregnant women with different demographic characteristics.

**Demographics**	**Total**	**High**	**Moderate**	**Low**	***χ^2^***	***P*-value**
	**(*n* = 973)**	**(*n* = 67)**	**(*n* = 200)**	**(*n* = 706)**		
Age, *M* (SD)	28.24 (4.88)	28.1 (4.09)	28.18 (4.44)	28.3 (5.06)	0.52	0.97
≤ 25	285 (29.29%)	18 (26.87%)	59 (29.50%)	208 (29.46%)		
26–34	572 (58.79%)	42 (62.69%)	116 (58.00%)	414 (58.64%)		
≥35	116 (11.92%)	7 (10.44%)	25 (12.50%)	84 (11.90%)		
**Education**						
High school or less	336 (34.53%)	25 (37.31%)	72 (36.00%)	239 (33.85%)	1.57	0.95
Universities and colleges	266 (27.34%)	17 (25.37%)	53 (26.50%)	196 (27.76%)		
Undergraduate	341 (35.05%)	24 (35.82%)	70 (35.00%)	247 (35.00%)		
Postgraduate	30 (3.08%)	1 (1.50%)	5 (2.50%)	24 (3.39%)		
**Only child**						
Yes	258 (26.52%)	15 (22.39%)	50 (25.00%)	193 (27.34%)	1.07	0.59
No	715 (73.48%)	52 (77.61%)	150 (75.00%)	513 (72.66%)		
**Residence**						
Urban	689 (70.82%)	48 (71.64%)	130 (65.00%)	511 (72.38%)	4.13	0.13
Rural	284 (29.18%)	19 (28.36%)	70 (35.00%)	195 (27.62%)		
**Monthly household income (RMB)**						
< 3,000	80 (8.22%)	3 (4.48%)	22 (11.00%)	55 (7.79%)	8.52	0.20
3,000–5,000	282 (28.98%)	27 (40.30%)	52 (26.00%)	203 (28.75%)		
5,001–7,000	243 (24.97%)	15 (22.39%)	45 (22.50%)	183 (25.92%)		
>7,000	368 (37.83%)	22 (32.83%)	81 (40.50%)	265 (37.54%)		
**Number of marriages**						
One marriage	918 (94.35%)	65 (97.01%)	191 (95.50%)	662 (93.77%)	1.84	0.40
Two marriages and above	55 (5.65%)	2 (2.99%)	9 (4.50%)	44 (6.23%)		
**Perinatal period**						
Second trimester	397 (40.80%)	20 (29.85%)	73 (36.50%)	304 (43.10%)	6.35	**0.04**
Third trimester	576 (59.20%)	47 (70.15%)	127 (63.50%)	402 (56.90%)		
**Marriage satisfaction**						
Satisfied	790 (81.19%)	45 (67.16%)	152 (76.00%)	593 (84.00%)	15.8	**< 0.001**
Dissatisfied	183 (18.81%)	22 (32.84%)	48 (24.00%)	113 (16.00%)		
**Conjugal relation**						
Good	902 (92.70%)	59 (88.10%)	180 (90.00%)	663 (93.91%)	5.81	0.06
Bad	71 (7.30%)	8 (11.90%)	20 (10.00%)	43 (6.09%)		
**Husband and wife status**						
Equality	863 (88.69%)	58 (86.57%)	172 (86.00%)	633 (89.66%)	2.41	0.30
Inequality	110 (11.31%)	9 (13.43%)	28 (14.00%)	73 (10.34%)		
**In-law relationship**						
Good	816 (83.86%)	43 (64.18%)	157 (78.50%)	616 (87.25%)	29.43	**< 0.001**
Bad	157 (16.14%)	24 (35.82%)	43 (21.50%)	90 (12.75%)		
**Friend relationship**						
Good	917 (94.24%)	56 (83.58%)	181 (90.50%)	680 (96.32%)	24.81	**< 0.001**
Bad	56 (5.76%)	11 (16.42%)	19 (9.50%)	26 (3.68%)		
**Basic diseases**						
Yes	315 (32.37%)	29 (43.28%)	75 (37.50%)	211 (29.90%)	8.04	**0.02**
No	658 (67.63%)	38 (56.72%)	125 (62.50%)	495 (70.10%)		
**Take birth control pills**						
Yes	343 (35.25%)	28 (41.79%)	86 (43.00%)	229 (32.44%)	8.97	**0.01**
No	630 (64.75%)	39 (58.21%)	114 (57.00%)	477 (67.56%)		

Bold values indicate *P* < 0.05.

The chi-square test results showed that participants reported differently in perinatal period, marriage satisfaction, in-law relationship, friend relationship, basic diseases, and taking birth control pills have different probabilities of being classified (all *P* < 0.05). No statistically significant differences of psychological symptoms were found in other variables.

### Multiple logistic regression analysis

Furthermore, focusing on the different profiles of the high symptoms group, the moderate symptoms group, and the low symptoms group, we used multiple logistic regression analysis to examine whether demographics predicted class membership, after adjusting for sociodemographic characteristic (age, education, only child, residence, and monthly household income).

**Figure 2:**
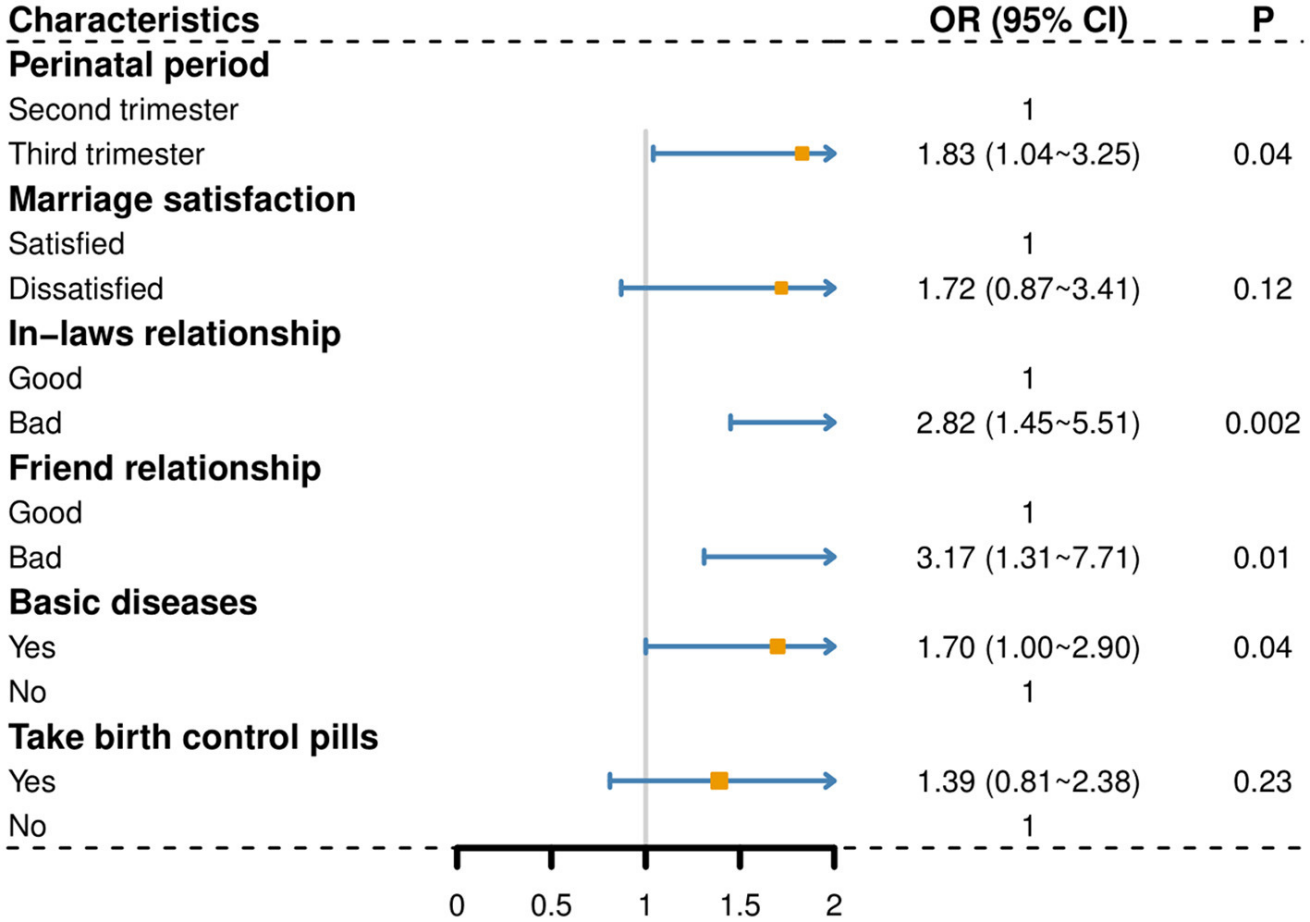
The high symptoms group compared to the low symptoms group (as a reference); A *P*-value of < 0.05 was considered significant.

First, the high symptoms group was compared with the low symptoms group (as a reference; Figure 2). Compared with the second trimester, the third trimester was more likely to be categorized into the high symptoms group (OR = 1.83, 95% CI: 1.04–3.25, *P* = 0.04). Compared with good in-law relationship, pregnant women with lousy in-law relationship exhibited higher odds of belonging to the high symptoms group (OR = 2.82, 95% CI: 1.45–5.51, *P* = 0.002). Compared with good friend relationship, those who had bad friend relationship were more likely to be categorized into the high symptoms group (OR = 3.17, 95% CI: 1.31–7.71, *P* = 0.01). Compared with no basic diseases, those who had basic diseases were easier to be divided into the high symptoms group (OR = 1.70, 95% CI: 1.00–2.90, *P* = 0.04).

**Figure 3:**
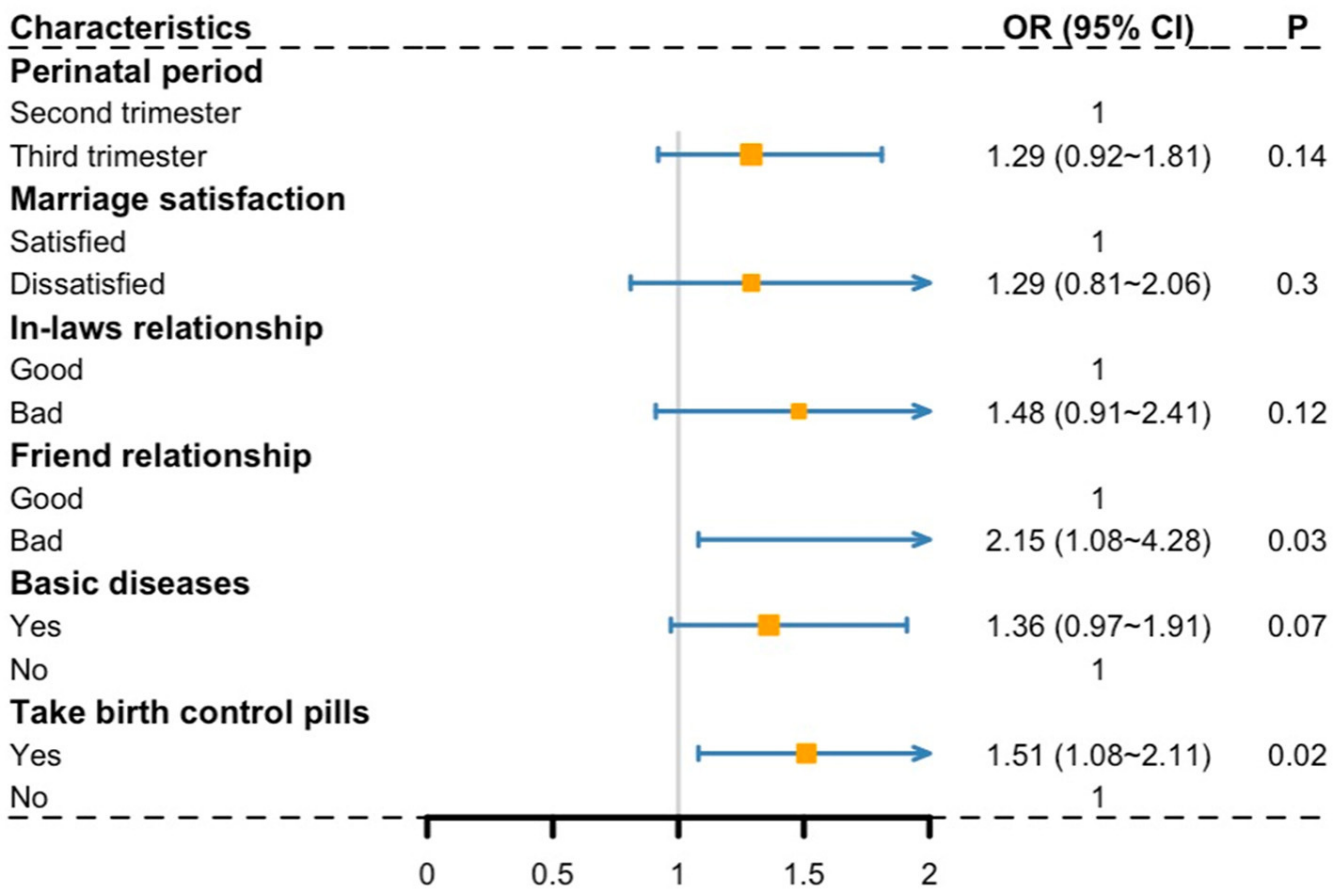
The moderate symptoms group compared to the low symptoms group (as a reference); A *P*-value of < 0.05 was considered significant.

Second, the moderate symptoms group was compared with the low symptoms group (as a reference) (Figure 3), those who have a bad friend relationship (OR = 2.15, 95% CI: 1.08–4.28, *P* = 0.03) and take birth control pills (OR = 1.51, 95% CI: 1.08–2.11, *P* = 0.02) were more likely divided into the moderate symptoms group.

## Discussion

In this study, we used the LCA to detect any causes that may result in significant heterogeneity of adverse psychological status among pregnant women. The LCA was selected over other exploratory approaches because it is model based, allows model comparisons to be statistically tested, and is appropriate for questionnaire-derived data, yet permitting differing variable measurement scales and variances (29, 34).

The findings of previous studies (35, 36) reported three or four latent classes, with one class having a low probability of psychological symptoms and the other having a high probability of psychological symptoms. The main findings of our study were as follows: (1) We identified three distinct subgroups, namely “low symptoms group,” “moderate symptoms group,” and “high symptoms group”; (2) different distributions during pregnancy were essential factors, and compared with the second trimester, participants in the third trimester were more likely to be categorized into the high symptoms group; (3) basic diseases or bad social relationships (including in-laws and friends) can also lead pregnant women to have an adverse psychological impact that leads them to be assigned to the high symptoms group; (4) the moderate symptoms group was more likely to have a bad friend relationship and take birth control pills than the low symptoms group.

### Effects of different stages of pregnancy

When compared to earlier findings (37, 38), heavier psychological symptoms are likely to occur in the third trimester than in the second trimester, which are consistent with the findings of this study. The reason for this development is attributed to the possibility that pregnant women are not satisfied with their body during pregnancy (39). From the second trimester to the third trimester, body dissatisfaction of pregnant women is positively correlated with psychological symptoms (40). Pregnant women with body dissatisfaction might fear negative evaluations by others and worry about their own ability to return to their pre-pregnancy body shape, which may predispose mental disorders (41). Moreover, according to a Korean study (42), psychological problems follow a growing pattern from the first to the third trimester. A systematic review (43) showed that second- and third-trimester cortisol assessments reported a connection, and elevated cortisol concentrations were observed in expected recovery periods. The higher the estradiol level in the third trimester, the greater the possibility of mental disorders. Therefore, medical institutions should take the screening of maternal mental health problems as a part of routine prenatal care (44). During each prenatal examination, they should ask about the emotional status of pregnant women and understand their psychosocial risk factors. It is important to provide standardized prenatal emotional management training for pregnant women (45). Noteworthy is that pregnant women in the third trimester are the potential population of postpartum depression (46). The mother, child, and family all have different challenges as a result of postpartum depression, which may also have an impact on the mother's ability to learn about baby care (47). Nursing staff have essential duties to carry out, including subjecting patients to efficient depression screenings and carrying out different approaches of psychological care according to the characteristics of different parturients, informing them about the possible postpartum emotional changes, giving appropriate encouragement or comfort, and guiding or helping them to improve their breathing patterns so as to eliminate the fear of the parturient.

### Underlying basic diseases or bad social environment

Our findings also revealed that pregnant women with basic diseases are apt to experience severer psychological symptoms. Psychological symptoms in the third trimester are related to the presence of pregnancy-related complications. Other authors (48, 49) have also found this relationship. Complications are difficult life experiences as well as a psychological burden for women. Moreover, pregnant women worry that their afflication of basic diseases will affect the health of their unborn child/children. In addition, bad social relationships (including in-laws and friends) are prone to worsen psychological symptoms. First of all, bad social relationships implies dangerous interpersonal relations, and by their nature, they may reduce social resources such as social support, and reduction in such social resources is a risk factor for mental health problems (50). Moreover, resource conservation theory (51) indicates that resource loss is the primary factor in the stress process as well as the cause of the emergence of psychological symptoms. Previous research (52) proved that the need for equal status as the husband in family relationships is one of the most influential factors causing mental disorders in pregnant women. Several lines of evidence (53–55) suggest that pregnant women may not be prepared to cope with the changes in social environments. Pregnant women are more likely to live in unstable social environments, lack security and support from their partners, and experience more marital conflict. Family members should give pregnant women full understanding and support and establish a good family support system. We should encourage tripartite talks among pregnant women, family members, and medical staffs during pregnancy to discuss how families should deal with common problems during pregnancy.

### The use of birth control pills

Some studies conducted in Western communities revealed a protective effect of birth control pills against mental health problems (56, 57). However, this study showed that taking birth control pills is a risk factor for psychological symptoms. A questionnaire study (58) of 101 birth control pill users and 90 pregnant controls revealed the presence of depression in 34%, irritability in 29%, adverse effects in 64%, etc. Having had a negative reaction to the confirmation of pregnancy was related to both probable depression and major depression in all three trimesters. A prospective cohort study (59) of 2,072 women was conducted in Sabah during 2009–2010, which suggested that pregnant women who were taking birth control pills appeared to have an increased risk of depression. As described earlier, birth control pills are likely to increase emotional lability, indicating that taking birth control pills will lead to heavier psychological symptoms.

As such, all these circumstances increase the risk of pregnant women's psychological symptoms.

### Strengths and limitations

Due to the complexity of the living environment and the differences between pregnant women (60), it is not convenient to simply classify individuals into whether they are in an adverse mood or not for researching pathogenesis. Thus, we explore the heterogeneity of perinatal adverse mood based on potential category variables from the pregnant respondents. The Chinese mental health scale was used to assess the adverse emotions of pregnant women, and patterns of psychological symptoms were identified to three classes by LCA, namely “high symptoms group,” “moderate symptoms group,” and “low symptoms group.” There were significant differences in 10 psychological items among different classes, which provide the convenience of implementation for investigating the relationship between the different demographic features and three latent phenotypes among Chinese pregnant women. Then, by analyzing the differences between different groups, we can find out the influencing factors that may lead to the bad mood of pregnant women.

However, the survey also has the following deficiencies and limitations. First, the sample distribution of the survey is not perfect, and the proportion of pregnant women in the high symptoms group is relatively small. Second, as a cross-sectional study, there was no follow-up survey on the production outcome and postpartum psychological status. Future research should involve larger well-designed studies, including different childbearing populations and postpartum follow-up surveys, to explore better the influencing factors of adverse emotions during pregnancy.

## Conclusions

The factors of pregnant women's mental health status include difficulty in the perinatal period, marital satisfaction, in-law relationship, friend relationship, basic diseases, and taking birth control pills. To ensure the smooth progress of pregnancy and promote the physical and mental health of pregnant women, psychological screening and psychological intervention measures should be strengthened.

## Data availability statement

The raw data supporting the conclusions of this article will be made available by the authors, without undue reservation.

## Ethics statement

The studies involving human participants were reviewed and approved by Ethics Committee of Xuzhou Medical University. The patients/participants provided their written informed consent to participate in this study.

## Author contributions

WW and XZ were involved in conceptualization and methodology. YP, QC, and YZ participated in data curation and writing—original draft preparation. JW, JT, HH, and ZZ supervised and validated the study. WW participated in writing—reviewing and editing. All authors contributed to the article and approved the submitted version.

## References

[1] Haque R Alam K Rahman SM Keramat SA Al-Hanawi MK. Women's empowerment and fertility decision-making in 53 low and middle resource countries: a pooled analysis of demographic and health surveys. BMJ Open. (2021) 11:e045952. 10.1136/bmjopen-2020-045952 34145014 PMC8215231

[2] Innocenti N Vignoli D Lazzeretti L. Economic complexity and fertility: insights from a low fertility country. Reg Stud. (2021) 55:1388–402. 10.1080/00343404.2021.1896695 34381283 PMC8300530

[3] Henriksson HE Malavaki C Bränn E Drainas V Lager S Iliadis SI. Blood plasma metabolic profiling of pregnant women with antenatal depressive symptoms. Transl Psychiatry. (2019) 9:204. 10.1038/s41398-019-0546-y 31444321 PMC6707960

[4] Kingston DE McDonald S Austin MP Hegadoren K Lasiuk G Tough S. The Public's views of mental health in pregnant and postpartum women: a population-based study. BMC Pregnancy Childbirth. (2014) 14:84. 10.1186/1471-2393-14-84 24564783 PMC3941946

[5] Bedaso A Adams J Peng W Sibbritt D. The association between social support and antenatal depressive and anxiety symptoms among Australian women. BMC Pregnancy Childbirth. (2021) 21:708. 10.1186/s12884-021-04188-4 34686140 PMC8532351

[6] Fuhler GM. The immune system and microbiome in pregnancy. Best Pract Res Clin Gastroenterol. (2020) 44–45:101671. 10.1016/j.bpg.2020.101671 32359685

[7] Tinius RA Yoho K Blankenship MM Maples JM. Postpartum metabolism: how does it change from pregnancy and what are the potential implications? Int J Womens Health. (2021) 13:591–9. 10.2147/IJWH.S314469 34168507 PMC8216742

[8] Wang X Yang T Miao J Liu H Wu K Guo J. Correlation between maternal and fetal insulin resistance in pregnant women with gestational diabetes mellitus. Clin Lab. (2018) 64:945–53. 10.7754/Clin.Lab.2018.171214 29945326

[9] Tinius RA Blankenship MM Furgal KE Cade WT Pearson KJ Rowland NS. Metabolic flexibility is impaired in women who are pregnant and overweight/obese and related to insulin resistance and inflammation. Metabolism. (2020) 104:154142. 10.1016/j.metabol.2020.154142 31930973 PMC7046129

[10] Putnam KT Wilcox M Robertson-Blackmore E Sharkey K Bergink V Munk-Olsen T. Clinical phenotypes of perinatal depression and time of symptom onset: analysis of data from an international consortium. Lancet Psychiatry. (2017) 4:477–85. 10.1016/S2215-0366(17)30136-0 28476427 PMC5836292

[11] Pampaka D Papatheodorou SI AlSeaidan M Wotayan RA Wright RJ Buring JE. Postnatal depressive symptoms in women with and without antenatal depressive symptoms: results from a prospective cohort study. Arch Womens Ment Health. (2019) 22:93–103. 10.1007/s00737-018-0880-8 29971553

[12] Kalmbach DA Cheng P Ong JC Ciesla JA Kingsberg SA Sangha R. Depression and suicidal ideation in pregnancy: exploring relationships with insomnia, short sleep, and nocturnal rumination. Sleep Med. (2020) 65:62–73. 10.1016/j.sleep.2019.07.010 31710876 PMC6980654

[13] Shay M MacKinnon AL Metcalfe A Giesbrecht G Campbell T Nerenberg K. Depressed mood and anxiety as risk factors for hypertensive disorders of pregnancy: a systematic review and meta-analysis. Psychol Med. (2020) 50:2128–40. 10.1017/S0033291720003062 32912348

[14] Glover V. Prenatal mental health and the effects of stress on the foetus and the child. Should psychiatrists look beyond mental disorders? World Psychiatry. (2020) 19:331–2. 10.1002/wps.20777 32931095 PMC7491637

[15] Beversdorf DQ Stevens HE Jones KL. Prenatal stress, maternal immune dysregulation, and their association with autism spectrum disorders. Curr Psychiatry Rep. (2018) 20:76. 10.1007/s11920-018-0945-4 30094645 PMC6369590

[16] Wiegersma AM Dalman C Lee BK Karlsson H Gardner RM. Association of prenatal maternal anemia with neurodevelopmental disorders. JAMA Psychiatry. (2019) 76:1294–304. 10.1001/jamapsychiatry.2019.2309 31532497 PMC6751782

[17] Maselko J Sikander S Bhalotra S Bangash O Ganga N Mukherjee S. Effect of an early perinatal depression intervention on long-term child development outcomes: follow-up of the Thinking Healthy Programme randomised controlled trial. Lancet Psychiatry. (2015) 2:609–17. 10.1016/S2215-0366(15)00109-1 26303558

[18] Mattina GF Van Lieshout RJ Steiner M. Inflammation, depression and cardiovascular disease in women: the role of the immune system across critical reproductive events. Ther Adv Cardiovasc Dis. (2019) 13:1753944719851950. 10.1177/1753944719851950 31144599 PMC6545651

[19] Bjelica A Cetkovic N Trninic-Pjevic A Mladenovic-Segedi L. The phenomenon of pregnancy - a psychological view. Ginekol Pol. (2018) 89:102–6. 10.5603/GP.a2018.0017 29512815

[20] Patten CA Lando HA Desnoyers CA Klejka J Decker PA Bock MJ. Association of tobacco use during pregnancy, perceived stress, and depression among alaska native women participants in the healthy pregnancies project. Nicotine Tob Res. (2020) 22:2104–8. 10.1093/ntr/ntz189 31566239 PMC7593352

[21] Maschke J Roetner J Bösl S Plank A-C Rohleder N Goecke TW. Association of prenatal alcohol exposure and prenatal maternal depression with offspring low-grade inflammation in early adolescence. Int J Environ Res Public Health. (2021) 18:7920. 10.3390/ijerph18157920 34360212 PMC8345560

[22] Popo E Kenyon S Dann SA MacArthur C Blissett J. Effects of lay support for pregnant women with social risk factors on infant development and maternal psychological health at 12 months postpartum. PLoS ONE. (2017) 12:e0182544. 10.1371/journal.pone.0182544 28846688 PMC5573293

[23] Sheeba B Nath A Metgud CS Krishna M Venkatesh S Vindhya J. Prenatal depression and its associated risk factors among pregnant women in bangalore: a hospital based prevalence study. Front Public Health. (2019) 7:108. 10.3389/fpubh.2019.00108 31131270 PMC6509237

[24] Joshi D Shrestha S Shrestha N. Understanding the antepartum depressive symptoms and its risk factors among the pregnant women visiting public health facilities of Nepal. PLoS ONE. (2019) 14:e0214992. 10.1371/journal.pone.0214992 30947251 PMC6448918

[25] Koelewijn JM Sluijs AM Vrijkotte TGM. Possible relationship between general and pregnancy-related anxiety during the first half of pregnancy and the birth process: a prospective cohort study. BMJ Open. (2017) 7:e013413. 10.1136/bmjopen-2016-013413 28490549 PMC5623367

[26] Ayaz R Hocaoglu M Günay T Yardimci OD Turgut A Karateke A. Anxiety and depression symptoms in the same pregnant women before and during the COVID-19 pandemic. J Perinat Med. (2020) 48:965–70. 10.1515/jpm-2020-0380 32887191

[27] Dong H Hu R Lu C Huang D Cui D Huang G. Investigation on the mental health status of pregnant women in China during the pandemic of COVID-19. Arch Gynecol Obstet. (2021) 303:463–9. 10.1007/s00404-020-05805-x 33009997 PMC7532741

[28] Lanza ST Rhoades BL. Latent class analysis: an alternative perspective on subgroup analysis in prevention and treatment. Prev Sci. (2013) 14:157–68. 10.1007/s11121-011-0201-1 21318625 PMC3173585

[29] Jisheng W. Compilation and standardization of Chinese adults' mental health scale. Chin J Public Health. (2006) 22:137–8. 10.3321/j.issn:1001-0580.2006.02.006

[30] Laska MN Pasch KE Lust K Story M Ehlinger E. Latent class analysis of lifestyle characteristics and health risk behaviors among college youth. Prev Sci. (2009) 10:376–86. 10.1007/s11121-009-0140-2 19499339 PMC2927491

[31] Nylund K Bellmore A Nishina A Graham S. Subtypes, severity, and structural stability of peer victimization: what does latent class analysis say? Child Dev. (2007) 78:1706–22. 10.1111/j.1467-8624.2007.01097.x 17988316

[32] Sun J-W Cao D-F Li J-H Zhang X Wang Y Bai H-Y. Profiles and characteristics of clinical subtypes of perinatal depressive symptoms: a latent class analysis. J Adv Nurs. (2019) 75:2753–65. 10.1111/jan.14136 31236991

[33] Carragher N Adamson G Bunting B McCann S. Treatment-seeking behaviours for depression in the general population: results from the National Epidemiologic Survey on Alcohol and Related Conditions. J Affect Disord. (2010) 121:59–67. 10.1016/j.jad.2009.05.009 19481816

[34] Haltigan JD Vaillancourt T. The influence of static and dynamic intrapersonal factors on longitudinal patterns of peer victimization through mid-adolescence: a latent transition analysis. J Abnorm Child Psychol. (2018) 46:11–26. 10.1007/s10802-017-0342-1 28861659

[35] Grunwell JR Gillespie S Morris CR Fitzpatrick AM. Latent class analysis of school-age children at risk for asthma exacerbation. J Allergy Clin Immunol Pract. (2020) 8:2275–84.e2272. 10.1016/j.jaip.2020.03.005 32198127 PMC7338257

[36] Haviland MJ Nillni YI Cabral HJ Fox MP Wise LA Burris HH. Adverse psychosocial factors in pregnancy and preterm delivery. Paediatr Perinat Epidemiol. (2021) 35:519–29. 10.1111/ppe.12756 33666948 PMC8380636

[37] Míguez MC Vázquez MB. Prevalence of depression during pregnancy in spanish women: trajectory and risk factors in each trimester. Int J Environ Res Public Health. (2021) 18:6789. 10.3390/ijerph18136789 34202666 PMC8297098

[38] Marchesi C Bertoni S Maggini C. Major and minor depression in pregnancy. Obstet Gynecol. (2009) 113:1292–8. 10.1097/AOG.0b013e3181a45e90 19461425

[39] Fuller-Tyszkiewicz M Skouteris H Watson BE Hill B. Body dissatisfaction during pregnancy: a systematic review of cross-sectional and prospective correlates. J Health Psychol. (2013) 18:1411–21. 10.1177/1359105312462437 23188921

[40] Chan CY Lee AM Koh YW Lam SK Lee CP Leung KY. Associations of body dissatisfaction with anxiety and depression in the pregnancy and postpartum periods: a longitudinal study. J Affect Disord. (2020) 263:582–92. 10.1016/j.jad.2019.11.032 31744745

[41] Fuller-Tyszkiewicz M Broadbent J Richardson B Watson B Klas A Skouteris H. network analysis comparison of central determinants of body dissatisfaction among pregnant and non-pregnant women. Body Image. (2020) 32:111–20. 10.1016/j.bodyim.2019.12.001 31855747

[42] Park JH Karmaus W Zhang H. Prevalence of and risk factors for depressive symptoms in korean women throughout pregnancy and in postpartum period. Asian Nurs Res. (2015) 9:219–25. 10.1016/j.anr.2015.03.004 26412626

[43] Nishi D Su K-P Usuda K Chang JP-C Hamazaki K Ishima T. Plasma estradiol levels and antidepressant effects of omega-3 fatty acids in pregnant women. Brain Behav Immun. (2020) 85:29–34. 10.1016/j.bbi.2019.02.014 30776476

[44] Barkin JL Van Cleve S. Screening for maternal mental health in the pediatric setting: situational stressors and supports. J Pediatr Health Care. (2020) 34:405–6. 10.1016/j.pedhc.2020.04.001 32414552

[45] Tschudin S Huang D Mor-Gültekin H Alder J Bitzer J Tercanli S. Prenatal counseling–implications of the cultural background of pregnant women on information processing, emotional response and acceptance. Ultraschall Med. (2011) 32(Suppl 2):E100–7. 10.1055/s-0031-1281665 22187410

[46] Mikšić Š Miškulin M Juranić B Rakošec Ž Včev A Degmečić D. Depression and suicidality during pregnancy. Psychiatr Danub. (2018) 30:85–90. 10.24869/psyd.2018.85 29546863

[47] Oztora S Arslan A Caylan A Dagdeviren HN. Postpartum depression and affecting factors in primary care. Niger J Clin Pract. (2019) 22:85–91. 10.4103/njcp.njcp_193_17 30666025

[48] Ajinkya S Jadhav PR Srivastava NN. Depression during pregnancy: prevalence and obstetric risk factors among pregnant women attending a tertiary care hospital in Navi Mumbai. Ind Psychiatry J. (2013) 22:37–40. 10.4103/0972-6748.123615 24459372 PMC3895310

[49] Thompson O Ajayi I. Prevalence of antenatal depression and associated risk factors among pregnant women attending antenatal clinics in Abeokuta North Local Government Area, Nigeria. Depress Res Treat. (2016) 2016:4518979. 10.1155/2016/4518979 27635258 PMC5007324

[50] Holmes EA O'Connor RC Perry VH Tracey I Wessely S Arseneault L. Multidisciplinary research priorities for the COVID-19 pandemic: a call for action for mental health science. Lancet Psychiatry. (2020) 7:547–60. 10.1016/S2215-0366(20)30168-1 32304649 PMC7159850

[51] Hobfoll SE. 127 Conservation of resources theory: its implication for stress, health, and resilience. In:Folkman S, editor. The Oxford Handbook of Stress, Health, and Coping. Oxford: Oxford University Press (2010), 127–47. 10.1093/oxfordhb/9780195375343.013.0007

[52] Wang J Pei Y Tang J Chen Q He C Zhang Y. The association between family relationships and depressive symptoms among pregnant women: a network analysis. Front Psychiatry. (2022) 13:919508. 10.3389/fpsyt.2022.919508 36072462 PMC9441886

[53] Sedgh G Bankole A Oye-Adeniran B Adewole IF Singh S Hussain R. Unwanted pregnancy and associated factors among Nigerian women. Int Fam Plan Perspect. (2006) 32:175–84. 10.1363/3217506 17237014

[54] Gariepy A Lundsberg LS Vilardo N Stanwood N Yonkers K Schwarz EB. Pregnancy context and women's health-related quality of life. Contraception. (2017) 95:491–9. 10.1016/j.contraception.2017.02.001 28188745 PMC5466832

[55] Shandley LM Kipling LM Spencer JB Morof D Mertens AC Howards PP. Factors associated with unplanned pregnancy among cancer survivors. J Womens Health. (2002) 31:665–74. 10.1089/jwh.2021.0176 34860591 PMC9133970

[56] Cheslack-Postava K Keyes KM Lowe SR Koenen KC. Oral contraceptive use and psychiatric disorders in a nationally representative sample of women. Arch Womens Mental Health. (2015) 18:103–11. 10.1007/s00737-014-0453-4 25113319 PMC4308571

[57] Keyes KM Cheslack-Postava K Westhoff C Heim CM Haloossim M Walsh K. Association of hormonal contraceptive use with reduced levels of depressive symptoms: a national study of sexually active women in the United States. Am J Epidemiol. (2013) 178:1378–88. 10.1093/aje/kwt188 24043440 PMC3888252

[58] Kane FJ Jr. Psychiatric reactions to oral contraceptives. Am J Obstet Gynecol. (1968) 102:1053–63. 10.1016/0002-9378(68)90471-7 5724394

[59] Mohamad Yusuff AS Tang L Binns CW Lee AH. Prevalence of antenatal depressive symptoms among women in Sabah, Malaysia. J Matern Fetal Neonatal Med. (2016) 29:1170–4. 10.3109/14767058.2015.1039506 26037724

[60] Howard LM Molyneaux E Dennis CL Rochat T Stein A Milgrom J. Non-psychotic mental disorders in the perinatal period. Lancet. (2014) 384:1775–88. 10.1016/S0140-6736(14)61276-9 25455248

